# Dissolution of an ensemble of differently shaped poly-dispersed drug particles undergoing solubility reduction: mathematical modelling

**DOI:** 10.5599/admet.841

**Published:** 2020-07-14

**Authors:** Michela Abrami, Lucia Grassi, Rosario di Vittorio, Dritan Hasa, Beatrice Perissutti, Dario Voinovich, Gabriele Grassi, Italo Colombo, Mario Grassi

**Affiliations:** 1 Dept. of Engineering and Architecture, Trieste University, Via Alfonso Valerio, 6/A, Trieste, I-34127 Italy; 2 Liceo Scientifico G. Galilei, Trieste, Via Mameli 4, I-34139 Italy; 3 Dept. of Chemical and Pharmaceutical Sciences, Trieste University, Piazzale Europa 1, Trieste, I-34127, Italy; 4 Dept. of Life Sciences, Cattinara University Hospital, Trieste University, Strada di Fiume 447, Trieste, I-34149 Italy

**Keywords:** Recrystallization, mathematical modelling, dissolution, particles, poly-dispersion, bioavailability

## Abstract

The aim of this theoretical paper is to develop a mathematical model for describing the dissolution process, in a finite liquid environment, of an ensemble of poly-dispersed drug particles, in form of sphere, cylinder and parallelepiped that can undergo solubility reduction due to phase transition induced by dissolution. The main result of this work consists in its simplicity as, whatever the particular particles size distribution, only two ordinary differential equations are needed to describe the dissolution process. This, in turn, reflects in a very powerful and agile theoretical tool that can be easily implemented in electronic sheets, a widespread tool among the research community. Another model advantage lies on the possibility of determining its parameters by means of common independent techniques thus enabling the evaluation of the importance of solid wettability on the dissolution process.

## Introduction

The great variety of controlled drug delivery systems is designed in reason of the drug to be delivered and the clinical target that, in turn, determine the choice of administration routes, among which the most important are the oral, injectable, inhaled, transmucosal, transdermal and the implantable one [1]. Despite the specific delivery system and the administration route considered, the *in vivo* drug fate is usually characterized by some common steps such as the release from the delivery system and the subsequent tissue absorption and distribution, metabolism and elimination (L-ADME processes) [2]. Specifically to the first step, the release kinetics can be ruled by different phenomena including swelling, erosion, drug dissolution, drug transport (due to diffusion and/or convection), drug interaction with the delivery system, initial drug distribution inside the delivery system and some delivery system geometrical characteristics [3, 4]. Independently from the administration route and drug, a key factor for the success and reliability of every delivery system is drug bioavailability, defined as the rate and extent to which the active drug is absorbed from a pharmaceutical form thus becoming available at the site of drug action [5, 6]. In turn, bioavailability depends strongly on drug permeability through cells membrane and drug solubilisation in physiological fluids. This is clearly pointed out by the bio-pharmaceutic classification according to which drugs can be subdivided into four classes: class I (high solubility and high permeability), class II (low solubility, high permeability), class III (high solubility, low permeability) and class IV (low solubility and low permeability) [7]. While permeation implies drug partitioning between a polar aqueous phase and an a-polar phase (cellular membranes), unless active mechanisms rule drug permeation, solubilisation implies the drug dissolution process. This phenomenon must not be confused with the release process as dissolution is defined as “the mixing of two phases with the formation of a new homogeneous phase (i.e. the solution)” [8]. Dissolution, in particular, assumes relevant importance for class II drugs that, interestingly, represent about 40% of the marketed drugs [9, 10] and 70-90% of the new chemical entities [9–12]. Indeed, most of the drugs are optimized solely on the basis of their pharmacological activity and not for what concerns bioavailability. Examples of commonly marketed drugs that are poorly soluble in water (less than 100 μg/cm^3^ [13]) include non-steroidal anti-inflammatory drugs (NSAIDs), anticholesterol, antimycotics, antibiotics, anticonvulsants, chemotherapeutics, antivirals, β-blockers, calcium channel blockers and immunosuppressants [4, 14-18].

The above considerations make clear why the drug dissolution process is actively studied in the pharmaceutical field from both an experimental and a theoretical viewpoint [4, 19]. Experimentalists, typically, prefer to perform Dissolution Rate Test (DRT) [20, 21], implying the dissolution of an ensemble of poly-dispersed drug particles in water or in a physiological fluid. In contrast, theorists propend for Intrinsic Dissolution Rate test (IDR), implying the dissolution from the flat surface of a drug tablet fixed to a rotating shaft [4]. While DRT is much closer to the *in vivo* drug performance, IDR is much simpler to be modelled. Indeed, IDR takes place in a much more controlled frame as fluid hydrodynamics around the flat rotating surface is well understood thanks to the elegant approach of Levich [22]. However, nowadays, the necessity of designing more and more sophisticated delivery systems stimulated theorists to move towards experimentalists. Therefore, interesting studies about the hydrodynamic conditions taking place in DRT [23, 24] and the direct observation of particles size reduction during DRT [19] have been undertaken. Additionally, due to the relevance in the pharmaceutical field, theorists started considering also possible variation of solubility occurring upon dissolution, whose kinetics, as later discussed, essentially depends on the surface available for dissolution, on mass transport resistance occurring at, and around, the solid/liquid interface and on drug solubility in the liquid phase. Indeed, it is well known that the contact between the drug and the liquid environment can lead to polymorphic transformations, as in the case of anhydrous theophylline that becomes hydrated [25] or nicergoline [26], or to amorphous drugs that recrystallize in the most stable crystalline form as it occurs for temazepam [14], nimesulide [27] and posaconazole [28]. Typically, phase transformations imply a reduction of solubility that, unavoidably, reflects in a reduction of drug bioavailability. Thus, these evidences underline the important role played by solubility on dissolution kinetics and increase the complexity of the already complex problem regarding solubility determination [29–31].

Historically, the first fundamental approach aimed at the description of DRT was that of Hixson and Crowell [32-34]. For the first time, they accounted for surface reduction upon dissolution of spherical particles and build up the famous cubic law. Then, the elegant model of Pedersen and co-workers accounted also for spherical particles poly-dispersion [35-38]. Interestingly, this model reduces to the Hixson-Crowell one in the case of monodispersed spherical particles. Since then, many other models were built up. Among others, we can remember the one of Thormann and co-workers that considers also the possible drug degradation after dissolution [39], the model of Hirai and co-workers that does not account explicitly for the shape of particles but focuses the attention on a law able to describe the time dependence of the dissolution surface [40] and the model of Guo and co-workers focusing on polymorphic transformation induced by dissolution (rifampicin, from form II to I) [41].

The aim of this paper is to build up a mathematical model able to merge a reasonably accurate description of the physical phenomena occurring in DRT with the practical need of experimentalists who demand theoretical tools able to guide experiments design and to allow a reliable data interpretation. In particular, the model will account for drug solubility reduction upon dissolution, particle poly-dispersion, particle geometry (spherical, cylindrical and parallelepiped) and the presence of a finite release environment.

## Mathematical Modelling

Dissolution can be considered as a consecutive process made up by five steps [8, 42, 43]: 1) contact of the solvent with the solid surface (*wetting*), which implies the production of a solid/liquid interface starting from solid/vapor one, 2) breakdown of intermolecular bonds in the solid phase (*fusion*), 3) molecules transfer from the solid phase to the solid/liquid interface (*solvation*), 4) diffusion of the solvated molecules through the unstirred boundary layer surrounding the solid surface (*diffusion*), 5) convective transport of solvated drug molecules into the well stirred bulk solution (*convection*). The first four steps, that can be viewed as the sum of four energy steps, represent the total resistance that the drug molecules have to overcome in order to move from the solid phase to the solution one (dissolution). Obviously, the higher the dissolution energy required (i.e. the higher the mass transfer resistance) the lower the dissolution kinetics is.

In order to connect the four steps, we need to know the time evolution of the drug concentration profile in the unstirred boundary layer surrounding the solid surface, whose presence is unavoidable and whose thickness depends on the relative velocity among particles and external fluid, on fluid kinematic viscosity, on particles dimension and on drug diffusion coefficient in the unstirred layer [24]. For this purpose, recourse can be made to the Fick’s second equation:


(1)

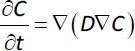



where *C* is drug concentration, *t* is time, *D* is the drug diffusion coefficient in the unstirred layer and ∇ is the gradient vector whose components analytical expression depends on the particular reference system chosen (Cartesian, spherical, cylindrical). The first model assumption relies on the hypothesis that mass transport inside the unstirred layer is one dimensional (the direction is that perpendicular to the solid surface) while the second one consists of the rapid attainment of pseudo-stationary conditions in the unstirred layer (this hypothesis is supported by the numerical solution of Eq. (1) assuming usual values for *D* (~ 10^–10^ m^2^ s^–1^) [4] and stagnant layer thickness ≤ 20 μm). Thus, Eq. (1) becomes:


(2)

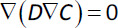



Eq. (2) has to be solved considering the following initial and boundary conditions:


(3)

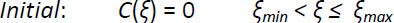




(4)

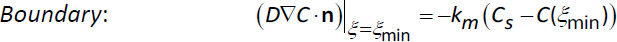




(5)

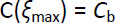



where ξ is the one dimensional spatial coordinate, (ξ_max_ -ξ_min_) is the thickness of the unstirred layer (δ), **n** is the surface normal versor, *C*_s_ is drug solubility in the dissolution liquid while *k*_m_ is the interface mass transfer coefficient mainly depending on the dissolution surface wetting properties and representing dissolution step 1. Eq. (3) affirms that, at the beginning, the unstirred layer does not contain drug molecules while Eq. (4) states that the drug flux leaving the solid surface depends on *k*_m_ and on the difference between drug solubility and drug concentration at the solid-liquid interface on the liquid side. Finally, Eq. (5) imposes that drug concentration equates *C*_b_ at the unstirred layer – bulk liquid interface. Eq. (2) solution, in Cartesian, cylindrical and spherical coordinates reads, respectively:


(6)

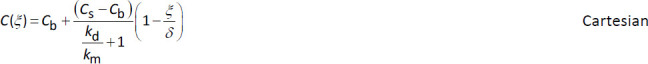




(7)

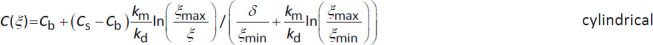




(8)

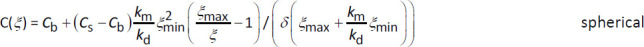



where *C*_b_ is the drug concentration in the bulk liquid at time *t*, *C*_s_ is drug solubility in the liquid phase and *k*_d_ is the mass transport coefficient (= *D*/*δ*) accounting for the fourth dissolution step (drug diffusion through the unstirred liquid layer). Eqs. (6) – (8) make clear that while in the Cartesian case (flat dissolution surface) a linear concentration profile develops in the unstirred layer, not linear concentration profiles take place when the dissolution surface is not flat. In addition, Eqs. (6) – (8) allow to evaluate the drug concentration *C*_0_ in ξ = ξ_min_, i.e., at the solid-liquid interface (liquid side):


(9)

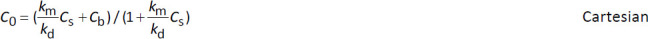




(10)

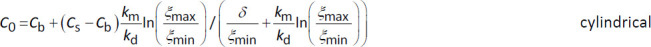




(11)

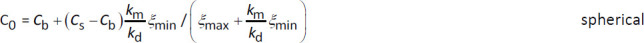



Eqs. (9) – (11) affirm that at *t* = 0, when *C*_b_ = 0, *C*_0_ is a fraction of *C*_s_, while *C*_0_ = *C*_s_ after a very long time when *C*_b_ = *C*_s_, i.e. *C*_0_ increases with time up to *C*_s_. When no wettability problems occur (*k*_m_ → ∞), *C*_0_ is always equal to *C*_s_ while it is equal to *C*_b_ = 0 when *k*_m_ → 0 (very poorly wettable solids).

By means of Eqs. (6) – (8), it is possible writing the ordinary differential equation accounting for drug concentration increase in the liquid phase:


(12)

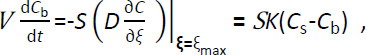



where *V* is the liquid volume and *K* is an overall mass transport coefficient assuming different analytical expressions depending on the particular coordinate system considered:


(13)

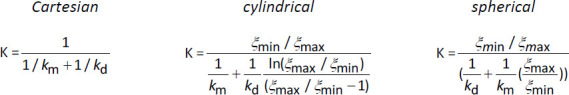



Eq. (12) states that the increase of the drug mass in the fluid phase is equal to the drug flow leaving the solid surface available for dissolution. In addition, Eq. (13) says that when no wettability problems arise  (*k*_m_ → ∞) *K* coincides with *k*_d_, provided that the thickness of the unstirred layer δ is very small (*ξ*_max_ ≈ *ξ*_min_) in the case of cylindrical and spherical geometry (obviously, when *k*_m_ → 0, *K* → 0). Although *K* varies with particle dimension as *k*_d_ depends on particle dimension [24], in order to simplify the scenario, the third hypothesis of the present model considers the *K* independence on particle dimensions so that an average value has to be considered. As a matter of fact, the great advantage of this usual hypothesis consists in an easy description of the dissolution process characterizing an ensemble of poly-dispersed particles of different shape (parallelepiped, cylinder, sphere). Indeed, it allows assuming that the solid amount dissolved per unit time and surface is the same whatever the surface delimiting the solid particle. Accordingly, in the case of a parallelepiped of dimensions *X*_i_, *Y*_i_, *Z*_i_, we have:


(14)

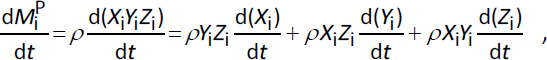




(15)

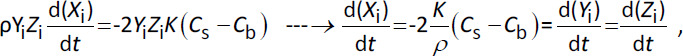



where *M*_i_^P^ and *ρ* are the parallelepiped mass and density, respectively. As the displacement Δ of the dissolution front is the same for each one of the parallelepiped surface, from Eq. (15) we have:


(16)

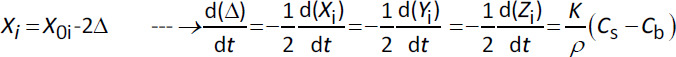



In the case of a cylinder of dimensions *R*_i_, *L*_i_, we have:


(17)

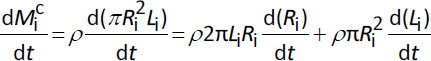




(18)

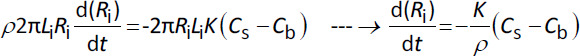




(19)

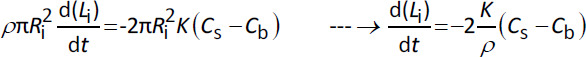



where *M*_i_^c^ is the cylinder mass. Consequently, it follows:


(20)

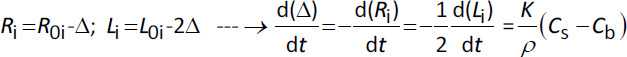



Finally, for a sphere of radius *R*_i_, we have:


(21)

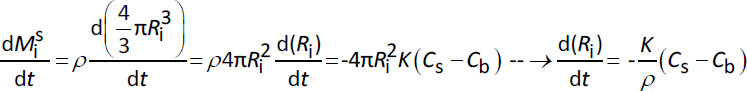



where *M*_i_^s^ is the sphere mass. Consequently, it follows:


(22)

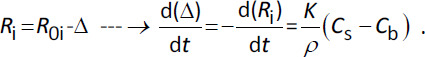



The most important message of Eqs. (14) – (22) relies on the possibility of determining particles dimensions upon dissolution, regardless of the geometry, by the evaluation of just the dissolution front displacement Δ.

In order to complete the model, it is necessary evaluating both *C*_s_ and *C*_b_. Indeed, as discussed in the introduction, it is quite common that, upon dissolution, the drug undergoes a phase transformation (polymorphic or amorphous – crystalline) implying a solubility reduction. Typically, this phenomenon is described by a first order reaction [44] occurring at the solid-liquid interface and leading to the following expression for the *C*_s_ temporal reduction:


(23)

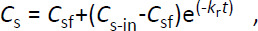



where *C*_sf_ and *C*_s-in_ are, respectively, the final and initial values of solubility while *k*_r_ is the recrystallization constant and *t* is time. As a matter of fact, Eq. (23) accounts for the dissolution step 2 as solubility is directly connected with the crystal network breakdown attitude that is quantified by its melting temperature and enthalpy [45]. In order to account also for a possible recrystallization in the bulk phase (occurring when *C*_b_(*t*) > *C*_s_(*t*)), it is necessary considering the following equation:


(24)

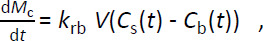



where *M*_c_ is the amount of recrystallized solid and *k*_rb_ is the bulk recrystallization constant that can differ from *k*_r_. Obviously, while Eq. (24) works only when *C*_b_(*t*) exceeds *C*_s_(*t*), the initial value for *M*_c_ is set to zero. The determination of *C*_b_ relies on a global mass balance ensuring that the initial solid mass (*M*_0_) must be equal, at any time, to the sum of the undissolved mass, the solubilized drug present in the bulk solution and *M*_c_ (the very small thickness of the boundary layer renders negligible the drug amount contained in it):


(25)

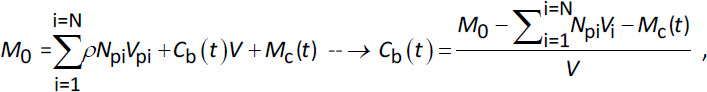



where *N*_pi_ represents the number of particles characterized by a volume *V*_pi_ and *N* is the number of classes into which the particle size distribution is subdivided in. It is interesting to notice that Eq. (25), de facto, accounts for the presence of a finite volume of the liquid phase (*V*). Indeed, when *V* → ∞, *C*_b_ will be always zero. For its mathematical attitude in describing particle size distributions, the Weibull equation was considered to describe the initial particles size distribution [46]:


(26)

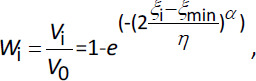



where *V*_0_ is the total volume of the solid drug, *V*_i_ is the volume occupied by all the particles sharing the same characteristic dimension *ξ*_i_ (radius in the case of spheres and cylinders, *X* dimension in the case of parallelepipeds), *ξ*_min_ is the minimum value of *ξ*_i_ while *η* and *α* are two model parameters. Once particle geometry is fixed, *V*_i_ enables the determination of *N*_pi_ by a simple geometric relation. Obviously, when the generic ξ_i_ goes to zero due to dissolution, *N*_pi_ is set to zero as this particle class has disappeared and it no longer contributes to the dissolution phenomenon. Thus, Eq. (26) serves only to evaluate the initial number of particles belonging to class i^th^.

In conclusion, the proposed model, establishing a connection among the dissolution steps, assumes that the dissolution kinetics can be essentially affected by steps 1, 2 and 4, attributing to step 3 a negligible role. In addition, whatever the number of classes constituting the particles size distribution, this model needs only two differential equations: one among the differential equations appearing in Eq. (16), (20) and (22), aimed at the prediction of the time evolution of the dissolution front position (Δ), plus Eq. (24) accounting for the time evolution of the recrystallized drug amount from the solution. Indeed, once Δ is known, it is possible evaluating the dimension *ξ*_i_ for all the particle class particles resorting to the algebraic relation linking *ξ*_i_ to Δ for the different geometries (see the first relation appearing in Eqs. (16), (20) and (22)). Of course, when *ξ*_i_ ~ 0 due to the dissolution process, the particle class particles is no longer considered letting *ξ*_i_ = *N*_pi_ = 0. As this model does not lead to an analytical solution, its iterative numerical solution (relaxation method, relaxation parameter *λ* = 1, relative tolerance = 10^-3^ [47]) was performed discretizing the two differential equations by means of the implicit Euler method. In order to ensure the numerical solution accuracy and stability, the time step was set to 0.25 s and the particles size distribution was subdivided into *N* = 200 classes.

## Results and Discussion

As many parameters affect model output, it would be impossible discussing the effects of all of them and how they interact to affect the dissolution kinetics. Accordingly, we decided to focus on three aspects, namely particle shape (parallelepiped, cylinder, sphere), particle size distribution and the ratio (*V*^+^) between the liquid phase volume (*V*) and the initial solid drug volume (*M*_0_/*ρ*). Indeed, especially the last two, represent the most important parameters available for the designing of the experimental set up.

**Figure 1.**
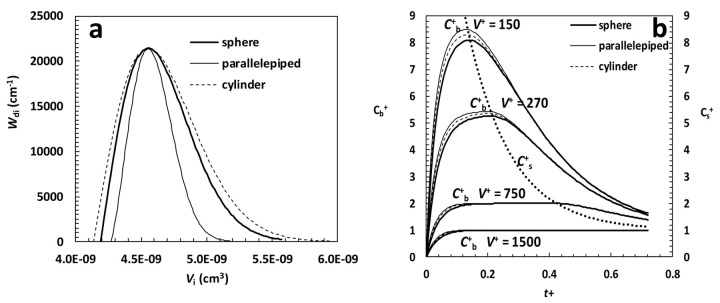
(a) Weibull differential distribution Wdi vs particles volume Vi for spheres, parallelepipeds and cylinders. Wdi parameters read: sphere – α = 2, η = 0.8 μm, Rmin = 10 μm -, parallelepiped - α = 2.35, η = 0.9 μm, Xmin = 16 μm, Fyx = Fzx = 1 (cubes) -, cylinder - α = 2, η = 0.8 μm, Rmin = 9 μm, FR = 2 (cubical cylinders). (b) Model predictions relative to the particle size distribution shown in Figure 1a. Cb+ (left vertical axis) and Cs+ (right vertical axis) are, respectively, the dimensionless drug concentration and solubility in the liquid phase (normalized with respect of the drug final solubility Cf) while t+ is a dimensionless time defined by 
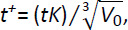
 being K the dissolution constant and V0 the particles volume before dissolution. V+ is the ratio between liquid volume (V) and V0. Other model parameters, set according to [4], read: ρ = 1.5 g/cm3, Cs-in = 2*10-2 g/cm3, Csf = 10-3 g/cm3, kr = krb = 10-2 s-1, K = 10-3 cm/s.

In order to properly evaluate the effect of particles shape on dissolution kinetics, the parameters of the Weibull distribution, whose differential expression is:


(27)

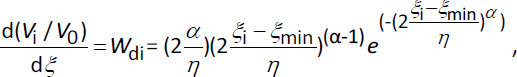



were fixed to get similar profile of Eq. (27) for parallelepiped, cylinder and sphere when plotted versus the volume competing to particles identified by the characteristic dimension *ξ*_i_. In the case of sphere *ξ*_i_ = *R*_i_ and the corresponding volume is (4/3)π*R*_i_^3^, in the case of cylinder *ξ*_i_ = *R*_i_ and the corresponding volume is π*R*_i_^3^*F*_R_, while, in the case of parallelepiped, *ξ*_i_ = *X*_i_ so that the corresponding volume is *X*_i_^3^*F*_yx_*F*_zx_. For the sake of simplicity, in the case of cylinders and parallelepipeds, it was assumed that the ratio *F*_R_ between cylinder radius and length, and the ratios *F*_yx_ = *Y*_i_/*X*_i_ and *F*_zx_ = *Z*_i_/*X*_i_ for parallelepiped were the same for all particles. Accordingly, *F*_R_, *F*_yx_ and *F*_zx_ can be considered as shape factors characterizing, respectively, cylinders and parallelepipeds. Figure 1a shows the trend of *W*_di_ for spheres, parallelepipeds and cylinders vs *V*_i_ assuming *F*_R_ = 2 and *F*_yx_ = *F*_zx_ = 1 (cubical cylinder and cube).

Although the three *W*_di_ do not share exactly the same wideness in *V*_i_, they are characterized by the same peak amplitude and position. In addition, as all of them are very narrow (10 μm ≤ *R*_i_ ≤ 11 μm, 16 μm ≤ *X*_i_ ≤ 17 μm and 9 μm ≤ *R*_i_ ≤ 10 μm for spheres, parallelepipeds and cylinders, respectively) they can be thought to be representative of approximately mono-dispersed size distributions. Relaying on these particle size distributions and assuming typical values for the other model parameters [4], Figure 1b shows model predictions considering different values of the ratio *V*^+^ between liquid volume (*V*) and initial particles volume (*V*_0_=*M*_0_/*ρ*) in the case of a drug undergoing recrystallization upon dissolution. It is clear that whatever *V*^+^, particle shape does not seem important as the dimensionless drug concentration in the liquid phase *C*_b_^+^ (= *C*_b_/*C*_f_; left vertical axis) is very similar for spheres (thick line), parallelepiped-cubes (thin line) and cubical cylinders (dashed line). In addition, when *V*^+^ is high (≥ 1500), the classical oversaturation peak does not appear (the over saturation condition is never met as *C*_b_^+^(*t*) is always lower that than *C*_s_^+^(*t*)) and only for smaller *V*^+^ it occurs, becoming evident for *V*^+^ ≤ 270. Finally, it is interesting to underline that the *C*_b_^+^ peak always occurs when the time dependent dimensionless drug solubility *C*_s_^+^ (= *C*_s_(*t*)/*C*_f_; right vertical axis) crosses the *C*_b_^+^ trend. Indeed, from now on, the solution is in over saturation conditions and only after a very long time *C*_b_^+^ equates *C*_s_^+^.

**Figure 2.**
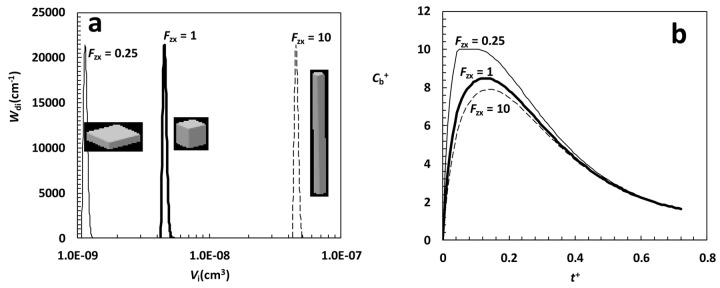
**(a)** Weibull differential distribution *W*_di_ *vs.* particles volume *V*_i_ for parallelepipeds characterized by three values of the *F*_zx_ shape factor (0.25, 1, 10), the same shape factor *F*_yx_ = 1 and the parameters adopted in Figure 1a (parallelepiped case): α = 2.35, η = 0.9 μm, *X*_min_ = 16 μm. **(b)** Model predictions relative to the particle size distribution shown in Figure 2a. *C*_b_^+^ is the dimensionless drug concentration in the liquid phase (normalized with respect of the drug final solubility *C*_f_) while *t*^+^ is a dimensionless time defined by , being *K* the dissolution constant and *V*_0_ the particles volume before dissolution. *V*^+^ (= 150) is the ratio between liquid volume (*V*) and *V*_0_. Other model parameters, set according to [4], read: ρ = 1.5 g/cm^3^, *C*_s-in_ = 2*10^-2^ g/cm^3^, *C*_sf_ = 10^-3^ g/cm^3^, *k*_r_ = *k*_rb_ = 10^-2^ s^-1^, *K* = 10^-3^ cm/s.

In order to appreciate the effect of particle shape factor on dissolution kinetics, we considered parallelepipeds characterized by three different values of the shape factor *F*_zx_ (0.25, 1 and 10), the same value for *F*_yx_ = 1 (parallelepipeds with square basis and different heights) and the same *X*_i_ size distribution adopted in Figure 1a (parallelepiped case). The three distributions considered, depicted in Figure 2a, clearly show that all other parameters being equal, the decrease of *F*_zx_ implies smaller particles and this, in turn, reflects in an increased dissolution surface. Practically speaking, we are comparing almost monodispersed particles size distributions composed by thin platelets (*F*_zx_ = 0.25), cubes (*F*_zx_ = 1) and long rods (*F*_zx_ = 10).

Assuming *V*^+^ = 150 and the same model parameters adopted in Figure 1b, model output is shown in Figure 2b. It is clear that, in this case, the effect of particle shape is no longer negligible as *C*_b_^+^ profile is undoubtedly affected by the increasing surface area available for dissolution induced by the different values of the shape factor *F*_zx_. Thus, particle shape factor becomes important as soon as it reflects in a significant variation of the area available for dissolution. In addition, it turns out that, in the case of drugs undergoing recrystallization, the characteristics of the oversaturation peak can also depend on particles shape factor.

**Figure 3.**
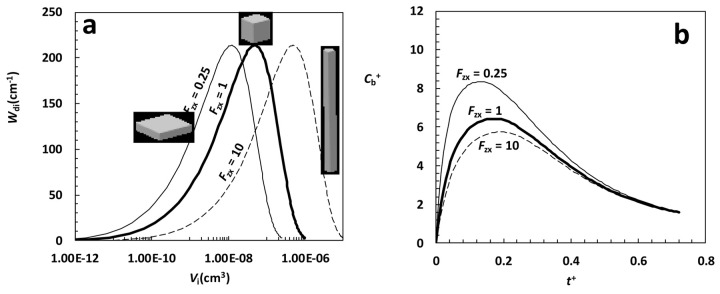
**(a)** Weibull differential distribution *W*_di_ vs particles volume *V*_i_ for parallelepipeds characterized by three values of the *F*_zx_ shape factor (0.25, 1, 10), the same shape factor *F*_yx_ = 1 and the following parameters: α = 2.35, η = 90 μm, *X*_min_ = 1 μm. **(b)** Model predictions relative to the particle size distribution shown in Figure 3a. *C*_b_^+^ is the dimensionless drug concentration in the liquid phase (normalized with respect of the drug final solubility *C*_f_) while *t*^+^ is a dimensionless time defined by , being *K* the dissolution constant and *V*_0_ the particles volume before dissolution. *V*^+^ (= 150) is the ratio between liquid volume (*V*) and *V*_0_. Other model parameters, set according to [4], read: ρ = 1.5 g/cm^3^, *C*_s-in_ = 2*10^-2^ g/cm^3^, *C*_sf_ = 10^-3^ g/cm^3^, *k*_r_ = *k*_rb_ = 10^-2^ s^-1^, *K* = 10^-3^ cm/s.

With the aim of exploring also the effect of particles poly-dispersion, Figure 3a considers parallelepipeds in form of platelets (*F*_zx_ = 0.25, *F*_yx_ = 1), cubes (*F*_zx_ = 1, *F*_yx_ = 1) and rods (*F*_zx_ = 10, *F*_yx_ = 1) characterized by the same poly-dispersion of the *X*_i_ dimension. With respect to the distributions shown in Figure 2a, the wideness of the three distributions is now increased as *X*_i_ spans from about 1 μm up to 100 μm while in Figure 2a we have 16 μm ≤ *X*_i_ ≤ 17 μm. Figure 3b witnesses that also in this case, despite poly-dispersion, a clear difference in the *C*_b_^+^ kinetics emerges as, again, platelets are characterized by the smallest particles and rods correspond to the biggest one while cubes put in the middle as shown by the position of each distribution peak. Obviously, the *C*_b_^+^ temporal evolution is a little bit depressed with respect to that reported in Figure 2b as poly-dispersion implies a reduction of the surface area available for dissolution. Again, the importance of poly-dispersion is strictly related to the effects it implies on the surface area available for dissolution.

### Case studies

After having explored some important model characteristics, with the aim to come closer to experimentalist need, in the following sections the attention will be focused on three different drugs undergoing solubility reduction upon dissolution.

**Figure 4.**
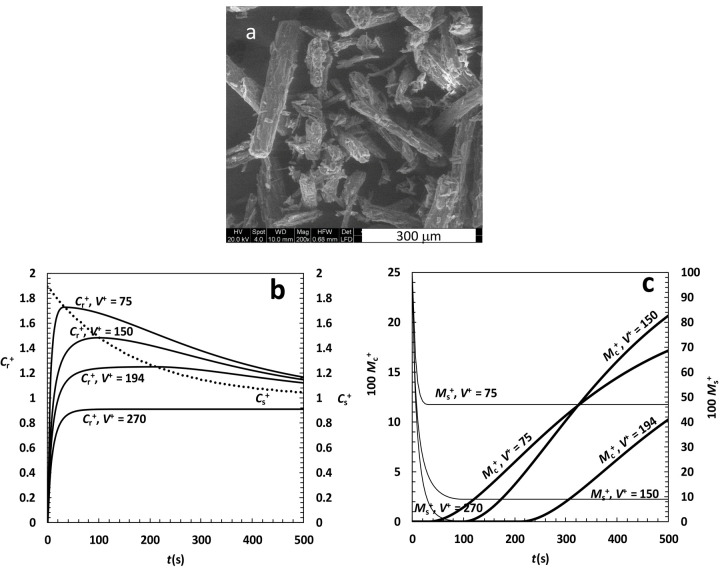
**(a)** Picture of anhydrous theophylline particles. **(b)** Model predictions about theophylline DRT assuming different values of the ratio *V*^+^ between liquid (*V*) and particles (*V*_0_) volume. *C*_b_^+^ (left vertical axis) and *C*_s_^+^ (right vertical axis) are, respectively, the dimensionless drug concentration and solubility in the liquid phase (normalized with respect to the drug final solubility *C*_f_). **(c)** Dimensionless amount of drug (*M*_s_^+^) still solid after time *t* and amount of recrystallized drug (*M*_c_^+^) in the liquid phase. Both amounts are normalized with respect of the initial drug particles mass *M*_0_.

The first drug considered is anhydrous theophylline (C_7_H_8_N_4_O_2_, M_w_ = 180.2; essentially neutral compound; it is a bronco-dilatator indicated mainly for asthma, bronchospasm, and COPD) that, upon dissolution in water, transforms into the more stable monohydrate form (C_7_H_8_N_4_O_2_^●^H_2_O, *M*_w_ = 198.2, *ρ* = 1.49 g/cm^3^ helium pycnometry) [25]. This polymorphic transformation implies a solubility reduction (*T* = 25 °C and pH = 7) from *C*_s-in_ = 11.6 mg/cm^3^ (anhydrous) to *C*_sf_ = 6.1 mg/cm^3^ (monohydrate). In addition, the analysis of IDR experiments reveals that *K* = 3.4*10^-3^ cm/s and *k*_r_ = 6*10^-3^ s^-1^ [4]. As one of the key factors ruling dissolution kinetics is the surface area of theophylline particles, particular care has to be devoted to the determination of this parameter. First of all, the inspection of Figure 4a suggests that rods (~ *F*_yx_ = *F*_zx_ = 0.25) could approximate the shape of anhydrous theophylline particles. Then, on the basis of this consideration, the parameters ruling the Weibull distribution, Eq. (27), are chosen in order to meet the experimentally determined theophylline specific area (0.331 m^2^/g, mercury porosimetry, unpublished data). The following values come out from this procedure: α = 2, η = 115 μm and 1 μm ≤ *X*_i_ ≤ 150 μm. Although this is not the unique choice of Eq. (27) parameters leading to the experimentally determined specific surface area, it surely represents a physically sound choice. Relying on this set of parameters, and assuming that the recrystallisation constant in the bulk liquid, *k*_rb_, equates that on particles surface (*k*_r_), model predictions about anhydrous theophylline dissolution are performed assuming different values of *V*^+^ as this is one of the most important and easy parameter to act on in order to properly design the experimental set up. Figure 4b indicates that the over saturation peak appears for *V*^+^ ≤ 194 and its amplitude increases with *V*^+^ reduction up to about 2, i.e. the value of the ratio *C*_s-in_/*C*_sf_. The decrease of the dimensionless solubility *C*_s_^+^, indicated by the dotted line in Figure 4b, always intersects the *C*_b_^+^ trend in its maximum and it is a measure of how fast the polymorphic transition takes place. In addition, Figure 4c allows to evaluate, for different *V*^+^ values, the temporal evolution of the dimensionless recrystallized drug amount (*M*_c_^+^) jointly with the dimensionless drug amount that has not yet undergone dissolution (*M*_s_^+^). While *M*_s_^+^ shows a faster decrease for increasing *V*^+^, less regular is the behaviour of *M*_c_^+^ due to its sigmoidal shape.

**Figure 5.**
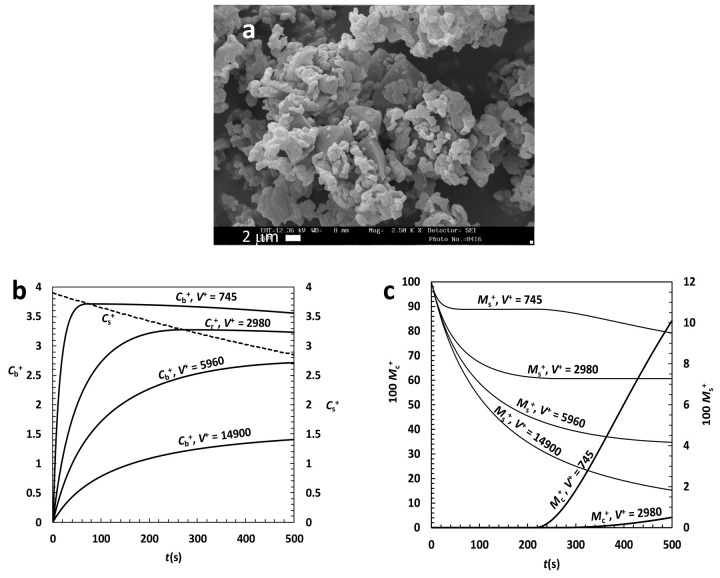
(a) SEM picture of griseofulvin particles. (b) Model predictions about griseofulvin DRT assuming different values of the ratio V+ between liquid (V) and particles (V0) volume. Cb+ (left vertical axis) and Cs+ (right vertical axis) are, respectively, the dimensionless drug concentration and solubility in the liquid phase (normalized with respect to the drug final solubility Cf). (c) Dimensionless amount of drug (Ms+) still solid after time t and amount of recrystallized drug (Mc+) in the liquid phase. Both amounts are normalized with respect of the initial drug particles mass M0.

The second drug considered is griseofulvin (C_17_H_17_ClO_6_, *M*_w_ = 352.8; *ρ* = 1.49 g/cm^3^ helium pycnometry; neutral compound; it is an antifungal drug used to treat a number of types of dermatophytoses), a typical class II drug that, upon milling, rapidly transforms in its amorphous state. The analysis of IDR tests reveals that, at 37 °C and pH = 7.5, the solubility of amorphous griseofulvin in water is 235 μg/cm^3^ while crystalline solubility is 60 μg/cm^3^. It is worth mentioning that 60 μg/cm^3^ exceeds the true griseofulvin solubility, i.e. that competing to griseofulvin crystals obtained by double recrystallization of native drug from acetonitrile, that is 12 μg/cm^3^. The reason for this discrepancy is due to the presence of many defects in the crystal structure of the commercially available griseofulvin so that it results to be a mixture of macrocrystals, nanocrystals and amorphous phase [4]. As it is well known that solubility increases with the reduction of crystal dimension [45], this discrepancy is not surprising. However, as, typically, DRT tests are performed on commercially available drugs, the solubility value of 60 μg/cm^3^ will be adopted for the following model simulations. Furtherly, the analysis of IDR test provides the following values for the dissolution *K* = 3.4*10^-3^ cm/s and the recrystallization *k*_r_ = 9*10^-4^ s^-1^ constants [4]. Again, the determination of the griseofulvin size distribution is performed by looking at the morphology of griseofulvin particles (Figure 5a) and, then, by choosing a reasonable set of Weibull parameters able to lead to the experimentally measured specific surface area (0.954 m^2^/g [48]).

Looking at Figure 5a, we can roughly assume that griseofulvin particles are cubes (*F*_yx_ = *F*_zx_ = 1) while a reasonable choice for the Weibull parameters is: α = 1.49, η = 13.6 μm and 0.1 μm ≤ *X*_i_ ≤ 100 μm. Relaying on this set of parameters, and assuming that the recrystallisation constant in the bulk liquid, *k*_rb_, equates that on particles surface (*k*_r_), model predictions about griseofulvin dissolution are performed assuming different values of *V*^+^ as this is one of the most useful parameters aimed at the proper experimental set up design. Figure 5b underlines that, due to relatively small *k*_r_ (= *k*_rb_) value (at least in comparison with that competing to anhydrous theophylline), the over saturation peak is scarcely visible, although present (see in Figure 5a the intersection of the various *C*_b_^+^ trends with *C*_s_^+^ trend), whatever *V*^+^. In this case, indeed, we should more properly speak about an “over saturation plateau” whose entity, obviously, increases with *V*^+^ decrease. A direct consequence of this behaviour is 1) the fast reduction of the drug amount that has not yet undergone dissolution (*M*_s_^+^) at time *t*, whose reduction kinetics increases with *V*^+^ (Figure 5c) and 2) the limited amount of the dimensionless recrystallized drug amount (*M*_c_^+^) that never exceeds the 10% of *M*_0_ even for the smallest *V*^+^ considered (Figure 5c).

The last drug studied is nimesulide (C_13_H_12_N_2_O_5_S, *M*_w_ = 308.5; *ρ* = 1.49 g/cm^3^ helium pycnometry; weak acid compound characterized by p*K*_a_ = 6.5 [49]; it is a non-steroidal anti-inflammatory drug), a typical class II drug that, upon milling, can be transformed in nanocrystals and amorphous state.

**Figure 6.**
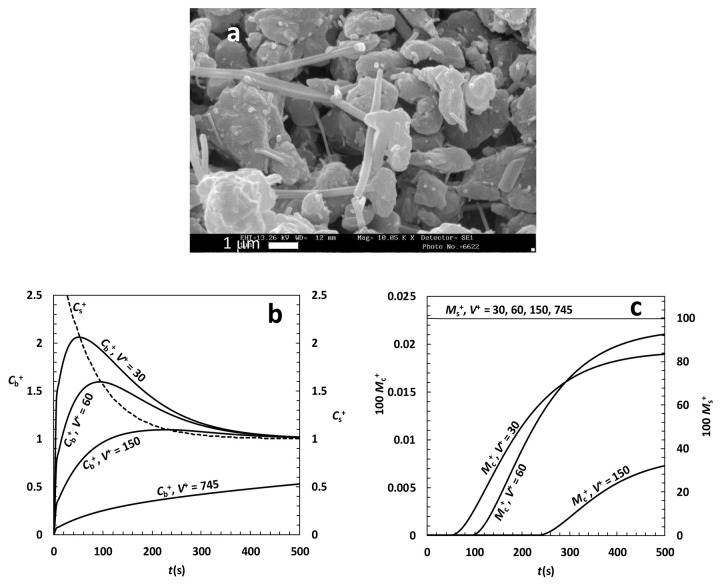
**(a)** SEM picture of nimesulide particles (needles) co-ground with polyvinylpyrrolidone (bigger particles). **(b)** Model predictions about nimesulide DRT assuming different values of the ratio *V*^+^ between liquid (*V*) and particles (*V*_0_) volume. *C*_b_^+^ (left vertical axis) and *C*_s_^+^ (right vertical axis) are, respectively, the dimensionless drug concentration and solubility in the liquid phase (normalized with respect to the drug final solubility *C*_f_). **(c)** Dimensionless amount of drug (*M*_s_^+^) still solid after time *t* and amount of recrystallized drug (*M*_c_^+^) in the liquid phase. Both amounts are normalized with respect of the initial drug particles mass *M*_0_.

Nimesulide is, among the three drugs considered, the less soluble in water being its solubility at 37 °C and pH ≤ 6 equal to 9 μg/cm^3^ and around 100 μg/cm^3^ at the same temperature but pH = 7.5 [50]. Fixing the attention on the pH ≤ 6 range, nimesulide solubility increases after co-grinding (in presence of a stabilizer) due to the transformation of macrocrystals into nanocrystals. Although solubility depends on nanocrystals dimension [45], we can set for nanocrystals solubility the value of 28 μg/cm^3^ that corresponds to four hours co-grinding as described in [27]. Figure 6a inspection reveals that nimesulide crystals look like needles so that their approximation by long cylinder (*F*_R_ ~ 15) seems the best choice. In order to match the experimentally determined specific area of nimesulide powder after grinding (2.7 m^2^/g, mercury porosimetry; unpublished data), a reasonable choice for the Weibull distribution is: α = 4, η = 0.54 μm and 0.3 μm ≤ *R*_i_ ≤ 0.8 μm. Finally, the analysis of the release test performed in [27] let to conclude that the dissolution *K* is equal to 1.8*10^-5^ cm/s and that the recrystallization constant *k*_r_ is equal to 1.3*10^-2^ s^-1^. If the small *K* value witnesses the very low nimesulide propensity to dissolve, this being due to its low wettability (static water contact angle ~ 70° [51]), the high *k*_r_ value indicates that nanocrystals and the amorphous phase are very instable and tend to rapidly recrystallize in the more stable crystalline form (this is the reason why nimesulide has to be co-ground together with a stabilizer, typically polyvinylpyrrolidone). Relaying on the above parameters set, and assuming that the re-crystallisation constant in the bulk liquid, *k*_rb_, equates that on particles surface (*k*_r_), model predictions about nimesulide dissolution are performed assuming different values of *V*^+^. Figure 6b shows the appearance of more and more pronounced oversaturation peak for reducing *V*^+^. In addition, the high *k*_r_ value (in comparison to those competing to theophylline and griseofulvin), makes peak shape very evident as the re-crystallization process is fast. Clearly, when *V*^+^ is sufficiently high, the peak disappears. The presence of a clearly identifiable peak, however, does not mean that a considerable amount of nimesulide goes in solution, as shown in Figure 6c. Indeed, dimensionless undissolved drug amount (*M*_s_^+^) is always close to 100% *M*_0_ and the dimensionless re-crystallized drug amount (*M*_c_^+^) is always lower that 0.025% *M*_0_, whatever *V*^+^ considered in the simulations.

## Conclusions

Assuming that:

1. mass transport inside the unstirred layer surrounding each particle is one dimensional,
2. that a rapid attainment of pseudo-stationary conditions in it takes place and that
3. the dissolution constant *K* is independent on particle dimensions,

the proposed model provides a description of the dissolution kinetics (DRT) from an ensemble of solid drug particles, eventually undergoing re-crystallization in a finite release environment, by means of only two differential equations, regardless of the number of the classes into which the continuous particle size distribution is subdivided. Obviously, this represents a considerable advantage in computational and practical terms as it allows to easily implement the model also by means of electronic sheets that are widespread in the research community. In addition, this model allows to select particle shape among spheres, cylinder (characterized by different length/radius ratios) and parallelepiped (characterized by different Y/X and Z/X ratios), this driving the model close to the real situation.

Despite this model requires a considerable number of parameters, the majority of them can be properly determined by means of common independent experiments (typically IDR test, mercury porosimetry, gas adsorption analysis (B.E.T.) and helium pycnometry) and only the dissolution constant *K* should be determined by data fitting. Indeed, as it depends on the relative velocity among particles and the surrounding liquid phase, its theoretical determination would require a complex analysis of the hydrodynamic conditions taking place in the liquid phase. In addition, we have to remember that *K* depends also on the mass transfer coefficient connected to the wettability of the solid surface (*k*_m_) (1/*K* is equal to the sum of the mass transfer resistances due to poor wettability (1/*k*_m_) and due to the presence of the unstirred layer surrounding each particle (1/*k*_d_)). Consequently, the *K* experimental determination is, in our opinion, the simplest way to proceed also because a proper experimental set up, implying DRT tests performed at increasing values of the Reynolds number (*Re*) (increasing with the stirring velocity imposed in the liquid phase), allows to separately evaluate *k*_d_ and *k*_m_. Indeed, for high *Re*, *K* should be almost independent on *Re* as *k*_d_ is proportional to *Re*^0.5^ [24] and, thus, d*k*_d_/d*Re* → 0 for high *Re*. Consequently, *K* should be mainly dependent on solid wettability 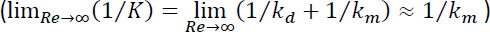. Once *k*_m_ is known, *k*_d_ could be determined in correspondence of the different *Re* considered in order to know the function *k*_d_(*Re*) as *k*_m_ is *Re* independent. Finally, *k*_m_ could be related to the work of solid immersion in the liquid phase.

In conclusion, this model allows to study and design DRT experiments permitting to enucleate the rate-determining steps ruling the entire phenomenon.
